# Hyaluronan injection in murine osteoarthritis prevents TGFbeta 1-induced synovial neovascularization and fibrosis and maintains articular cartilage integrity by a CD44-dependent mechanism

**DOI:** 10.1186/ar3887

**Published:** 2012-06-21

**Authors:** Jun Li, Daniel J Gorski, Wendy Anemaet, Jennifer Velasco, Jun Takeuchi, John D Sandy, Anna Plaas

**Affiliations:** 1 Department of Internal Medicine (Rheumatology), Rush University Medical Center, 1611 West Harrison Street Suite 510, Chicago, IL 60612, USA; 2 Department of Biochemistry Rush University Medical Center, 1735 W Harrison Street, Chicago, IL 60612, USA; 3 School of Physical Therapy, Carrol Hall, 421, Regis University 3333 Regis Blvd., Denver, CO 80221, USA; 4 Pharmaceuticals Information Group, Seikagaku Corporation, Marunouchi Center Building 6-1, Marunouchi 1-chome Chiyoda-ku Tokyo 100-0005, Japan

## Abstract

**Introduction:**

The mechanism by which intra-articular injection of hyaluronan (HA) ameliorates joint pathology is unknown. Animal studies have shown that HA can reduce synovial activation, periarticular fibrosis and cartilage erosion; however, its specific effects on the different cell types involved remain unclear. We have used the TTR (TGFbeta1 injection and Treadmill Running) model of murine osteoarthritis (OA), which exhibits many OA-like changes, including synovial activation, to examine *in vivo *tissue-specific effects of intra-articular HA.

**Methods:**

The kinetics of clearance of fluorotagged HA from joints was examined with whole-body imaging. Naïve and treated knee joints were examined macroscopically for cartilage erosion, meniscal damage and fibrosis. Quantitative histopathology was done with Safranin O for cartilage and with Hematoxylin & Eosin for synovium. Gene expression in joint tissues for *Acan*, *Col1a1, Col2a1, Col3a1*, *Col5a1, Col10a1, Adamts5 *and *Mmp13 *was done by quantitative PCR. The abundance and distribution of aggrecan, collagen types I, II, III, V and X, ADAMTS5 and MMP13 were examined by immunohistochemistry.

**Results:**

Injected HA showed a half-life of less than 2 h in the murine knee joint. At the tissue level, HA protected against neovascularization and fibrosis of the meniscus/synovium and maintained articular cartilage integrity in wild-type but not in *Cd44 *knockout mice. HA injection enhanced the expression of chondrogenic genes and proteins and blocked that of fibrogenic/degradative genes and proteins in cartilage/subchondral bone, whereas it blocked activation of both groups in meniscus/synovium. In all locations it reduced the expression/protein for *Mmp13 *and blocked *Adamts5 *expression but not its protein abundance in the synovial lining.

**Conclusions:**

The injection of HA, 24 h after TGFbeta1 injection, inhibited the cascade of OA-like joint changes seen after treadmill use in the TTR model of OA. In terms of mechanism, tissue protection by HA injection was abrogated by *Cd44 *ablation, suggesting that interaction of the injected HA with CD44 is central to its protective effects on joint tissue remodeling and degeneration in OA progression.

## Introduction

The generally accepted, albeit limited, benefit of hyaluronan (HA) injection for patients with osteoarthritis (OA) [1] has been accompanied by basic research, initiated in about 1996 [2], to unravel the mechanism(s) of this effect. Studies in OA models in rats, rabbits, dogs and sheep have indicated that HA has pleitrophic effects, such as anti-apoptotic, anti-inflammatory, anti-angiogenic and anti-fibrotic. For example, HA treatment of rats after joint immobilization [3] or intra-articular IL-1 injection [4] protects against cartilage degeneration, apparently due to both anti-apoptotic and anti-inflammatory effects. Moreover, OA-like changes after ovine anterior cruciate ligament transection (ACLT) or meniscectomy include fibrosis and neovascularization of the synovium, and this pathology is also ameliorated by HA injections [5,6]. In the same context, extended strenuous uphill running of rats [7] results in a fibrous deposition in the infrapatellar fat pad and this is prevented by HA injection during the exercise period. These inhibitory effects of HA on fibroplasia in animal joint tissues appear to be very relevant to human treatments since human OA has been associated with activation of pro-fibrogenic genes in cartilage [8,9] and overt fibrosis of the synovium [10-12], subchondral bone [13,14] and vastus medialis muscle [15].

We have reported that for mice, intra-articular injections of TGFbeta1 prior to treadmill running (TTR model) results in mechanically-induced fibrotic remodeling and erosion of the articular cartilage as well as synovial hyperplasia and fibrosis [16]. Notably, these pathologies did not develop in ADAMTS5-deficient mice, apparently because the absence of ADAMTS5 can prevent TGFbeta1-induced fibrogenesis (via Smad2/3), and promote TGFbeta1/BMP-induced chondrogenesis (via Smad1/5/8), a switch which has been demonstrated in newborn fibroblasts [17] and bone marrow derived mesenchymal stem cells (MSCs) (Gorski D and Plaas A, unpublished). Further, the chondrogenic effect of *Adamts5 *ablation in dermal fibroblasts *in vivo *was shown to be eliminated, and fibrogenic pathways activated by concomitant ablation of *Cd44 *[17].

Our primary goal in the current work was to use this murine model of OA to determine whether HA injection abrogates the fibrogenic cell and tissue changes which occur in the synovium/meniscus and cartilage/subchondral bone compartments in this model. As part of this objective we also studied the effect of HA injection on the expression and abundance of the two metalloproteases, ADAMTS5 and MMP13, which are now primarily invoked to explain tissue degeneration in murine and human OA [18]. In addition, studies in isolated chondrocytes and synoviocytes [19-22] have suggested a role for CD44 in HA-mediated inhibition of expression of MMP1/13 and ADAMTS4/5 by these cell types. To determine whether CD44 might also be required for the effects of HA *in vivo*, we have used *Cd44*-/- mice in the TTR model of OA.

## Materials and methods

### Osteoarthritis model and experimental groups

**Figure 1:**
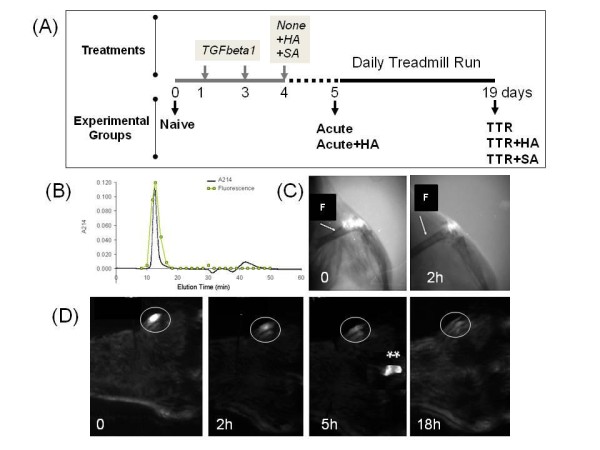
**Experimental protocols and elimination of HA from the murine knee joint**. (**A**) The schematic shows details of the time line for intraarticular injections, treadmill running and time points for tissue collection. (**B**) Characterization of FITC-labeled HA preparation (620 to 1,170 kDa) by Sephacryl S-1000 chromatography. (**C**) Fluorescence imaging and x-ray analyses of joints shows accuracy of delivery of FITC-labeled HA into the joint space. (F, Femur) (**D**) Whole body fluorescent imaging was used to determine the time course of disappearance from the joint of FITC-labeled HA (FITC labeled excretory products at 5 h after injection are indicated by **). Procedures are described in detail in the Methods section.

The murine TTR (TGFbeta1 injection plus Treadmill Running) model is described in detail elsewhere [16] and also summarized in Figure 1A. Male mice (wild type and *Cd44-/- *strains [17] in the C57BL/6 background, age 12 weeks) were bred in-house, and all animal protocols were approved by the Rush University Medical Center Animal Care and Use Committee. HA or saline injections were given one day after the second TGFbeta1 injection, and the effect was studied for acute changes and longer term changes (see Figure 1A for group names). For the acute study, the groups included: a) untreated mice (Naïve), b) mice sacrificed two days after the second TGFbeta1 injection without further treatment (Acute), and c) mice sacrificed one day after HA injection (Acute +HA). For the longer-term study, the three groups were: a) mice sacrificed on Day 19 after the two TGFbeta1 injections and 14 days treadmill running (TTR), b) mice sacrificed on Day 19 after TGFbeta1 treatment, HA injection and 14 days of treadmill running (TTR+HA) and mice sacrificed on Day 19 after TGFbeta1 treatment, saline injection and 14 days of treadmill running (TTR+SA). For the study with *Cd44-/- *mice, the groups analyzed were Naive, TTR and TTR+HA.

### Hyaluronan preparations, intra-articular delivery and dwell time

HA (Supartz, Seikagaku Co., Tokyo, Japan 620-1170 kDa, average 900 kDa) was diluted with sterile saline and 7.5 ug in 10 ul was injected through the patellar ligament into the joint space of the right knee with a 30-gauge needle on an insulin syringe. Fluorescein isothiocyanate (FITC)-labeled HA [23] was chromatographed on Sephacryl S-1000 (GE Healthcare Biosciences, Piscataway, NJ, USA) in 0.1 M sodium acetate, pH 7.0 [24] and the eluant monitored for fluorescence. Essentially all of the fluorescence was in high molecular weight HA (absorbance at 214 nm, A214) as shown by exclusion from the gel (Figure 1A). To check the accuracy of injection and the dwell time of HA in the joint, the FITC-labeled HA was injected into the knee of naïve mice (*n *= 4) and also mice which had been injected into the knee 24 h earlier with 200 ng active TGFbeta1 (*n *= 4). At 0, 2, 5 and 18 h, mice were placed in a Kodak FX System (Carestream Health Inc., Rochester, NY, USA) for X-ray and fluorescence analysis. Accuracy of injection was confirmed (Figure 1C) and the bulk of the injected HA were eliminated (from both naïve and TGFbeta1 injected joints) in the first 2 h, although traces could be detected at 18 h (Figure 1D). High fluorescence was also seen in the urethra at 5 h (Figure 1D), probably derived from the short-lived HA pool released from the joint in the first 2 h.

### Macroscopic imaging and histopathology

**Figure 2:**
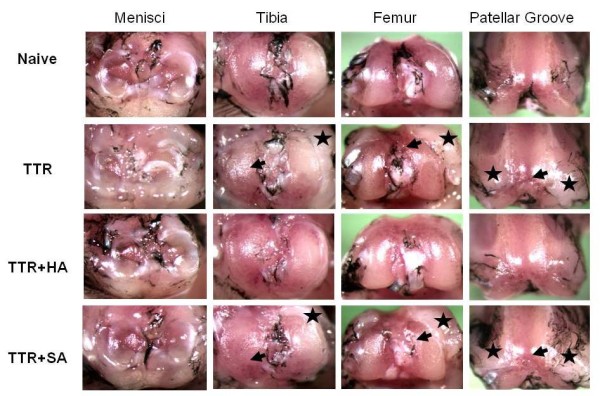
**Effect of HA injection on macroscopic pathology in the TTR model**. Knee joints were carefully opened, and after rinsing with PBS, India ink was applied to all surfaces with a paint brush and rinsed again. Menisci and articular surfaces were photographed under a Nikon dissecting microscope (SMZ1000, X6) and images processed with Spot Basic, Diagnostic Instruments, Inc (Sterling Heights, MI, USA). Typical images of meniscus and cartilage surfaces from naïve joints and joints from each of the three experimental groups at Day 19 (TTR, TTR+HA and TTR+SA) are shown. Black arrow heads indicate cartilage erosion and black stars indicate fibrotic overgrowth.

Global joint pathology [25] was evaluated by India Ink application followed by surface photography under a Nikon dissecting microscope (SMZ1000, X6) (Nikon Instruments Inc., Melville, NY, USA). Abnormalities were evaluated blindly, paying particular attention to the deposition of fibrotic tissue around the menisci and along the medial and lateral aspects of the tibial plateau, femoral condyles and patella groove margins, and any evidence of an associated cartilage surface roughening or erosion (see Figure 2). The reproducibility and discriminatory power of macroscopic evaluation was established earlier [25,26]; however, scoring was not used here because of the obvious and marked effects of HA on global pathology, histopathology and immunohistochemistry.

For histology, intact mouse knees were dissected away from the skin, fixed with 10% neutral buffered formalin for a minimum of three days, and decalcified in 5% ethylenediaminetetraacetic acid (EDTA)/phosphate-buffered saline (PBS) for three weeks. Specimens were paraffin embedded and about 180 thin sections (6 um) were taken across the entire joint from medial to lateral in the sagittal plane. Slides 1 to 30, 31 to 60 and 61 to 90 (two adjacent sections per slide) spanned the medial, central groove and lateral compartments, respectively. For histopathological assays, deparaffinized slides 1, 11, 21, 31, 41, 51, 61, 71 and 81 were stained with Safranin O and slides 2, 12, 22, 32, 42, 52, 62, 72, and 82 were stained with hematoxylin/eosin.

**Figure 3:**
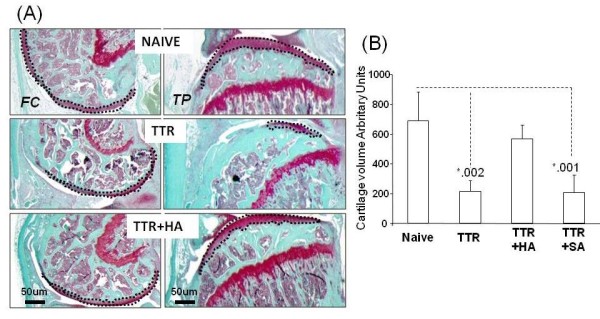
**Effect of HA injection on cartilage histopathology in the TTR model**. (**A**) Typical Safranin O histology of femoral and tibial epiphyses from naïve joints and joints of TTR and TTR+HA groups (**B**). For each of the experimental groups, the mean (+/SD) cartilage volume was calculated from the area stained with Safranin O as described in the Methods. The dotted lines show typical selections of areas used for cartilage quantitation.

The mean stainable cartilage in joints from each experimental group was determined as follows: the area (tibia plus femur) of Safranin O positive tissue was obtained by visual tracing (see Figure 3A), coupled to the area function of the NIH image analysis software Image J (Bethesda, MD, USA). Areas were determined on each of 32 equally spaced sagittal sections taken across the lateral compartment of the right joint of each mouse. The total area for each mouse was the summed area values of the 32 sections. The mean total area (+/-SD) (cartilage volume (arbitrary units) on Figure 3B) was calculated from the total area for each mouse (*N *= 6) in each group.

Synovial histopathology was scored essentially as described [5] on a scale of 0 to 5 for sub intimal fibrosis and for vascularity. The analysis was done on 16 equally spaced sagittal sections from the lateral compartment of each joint stained with hematoxylin/eosin, and only matching regions of the proximal and distal perimeniscal synovium were scored. The mean score for fibrosis or vascularity for each mouse was the sum of the scores from the 16 sections divided by 16. The mean scores (+/-SD) for fibrosis or vascularity in each group (*N *= 4) were calculated from the mean score from each mouse in the group.

### Quantitative PCR

A total of 16 naïve mice and 24 experimental mice for each treatment group (TTR, TTR+HA and TTR+SA) were analyzed as follows: articular surfaces from two mice were combined for each assay and these pools (*n *= 8 to 12) were analyzed separately. Cartilage-rich tissue was pooled from tibial and femoral surfaces by a fine scalpel cut across the surfaces. Histological inspection showed that all cartilage samples contained subchondral bone, but no growth plate cartilage, so that these samples are described as cartilage/subchondral bone throughout. The menisci and synovial tissue were harvested. This was done by making a circular incision along the synovium/periarticular attachments on the medial and lateral tibial plateaus, followed by cutting the anterior and posterior attachments of both menisci. For menisci/synovial tissue analysis, two tissue pools from each experimental group were prepared, with each derived from 8 to 12 mice. This was necessitated by the relatively low content/yield of mRNA from meniscus/synovium relative to cartilage/subchondral bone samples.

All specimens were harvested into RNALater and stored at -20C before analyses. RNA was prepared by thawing tissues on ice, rinsing with fresh RNALater (Qiagen, Valencia, CA, USA), snap-freezing in liquid nitrogen and pulverizing, prior to application of the PerfectPure RNA Kit for Fibrous Tissue (5 PRIME). Taqman-based QPCR (Life Technologies, Carlsbad, CA, USA) was done with inventoried primers for mouse *Acan*, *Col3a1 *and *Adamts5 *as described [17]. Primers for *Col1a1, Col5a1, Col10a1*, and *Mmp13 *were Mm00801666_g1, Mm00489342_m1, Mm004 87041_m1 and Mm00439491_m1, respectively. QPCR values of meniscus/synovium used for comparisons between experimental groups were the average of the data from the two pools, with the difference between the results being < 20% of the average pool value.

### Immunohistochemistry

Anti-collagen type I (ab34710), anti-collagen type II (ab34712), anti-collagen type III (ab7778), anti-collagen type V (ab7046), anti-collagen type × (ab58632) and anti-MMP13 (ab75606) were purchased from Abcam UK (Cambridge, MA, USA). Aggrecan and ADAMTS5 were detected as described [27] using anti-DLS and anti-KNG respectively. For each joint, the two sections on slides 3 to 10, 13 to 20, 23 to 30, 63 to 70, 73 to 80 and 83 to 90 were taken for immunohistochemistry. Briefly, deparaffinized sections were incubated in primary antibodies or non-immune IgG (both 10 ug/ml) overnight at 4°C. Sections to be stained with anti-MMP13, anti-ADAMTS5, anti-collagen type X and anti-collagen type II were digested with proteinase K to obtain optimal antigen exposure. Sections were next incubated with biotinylated goat anti-rabbit IgG, HRP-labeled avidin-biotin complex and 3, 3'-diaminobenzidine substrate. Nuclei and cartilage matrix were counter-stained with methyl green as described by Vector Labs, Burlingame, CA, USA. It should be noted that this IHC staining procedure predominantly stained antigens in the pericellular/cell-associated space and that antigen retrieval procedures, such as proteinase K, chondroitinase or hyaluronidase pretreatment, but did not significantly enhance general matrix staining. Further, all antibodies were shown to exhibit high specificity as determined with controls employing only the secondary antibody. Since the differences in signal intensity and distribution (with multiple antibodies on multiple sections) between treatment groups were highly reproducible and clearly biologically relevant, no scoring system or statistical evaluation was developed for this analysis.

### Statistical analysis

For those experiments where data were obtained separately from six or more individual mice (N > 5), two-way ANOVA for independent samples was used as an initial analysis, followed by Students *t*-test for comparisons between the most relevant pairs of groups. For menisci/synovial tissue no statistical analysis was applied.

## Results

### Effect of HA injection on macroscopic pathology and cartilage loss in the TTR Model

A schematic describing the time line of the model, treatment for each experimental group and tissue harvest points is shown in Figure 1A. The macroscopic pathology seen on the surfaces of menisci, tibia, femur and the patellar groove in the TTR model (Figure 2, Row 2), relative to the appearance of naive joints (Figure 2, Row 1), is shown. As described previously [16,25] in this model, there is a deposition of fibrotic tissue around the menisci and along the medial and lateral aspects of the tibial plateau, femoral condyles and patella groove margins, which is most likely derived from activation of synovium and periosteum by TGFbeta1 injection. Furthermore, this remodeling is commonly associated with cartilage surface roughening or erosion.

The effects of HA injection (TTR+HA) relative to saline injection (TTR+SA) is illustrated in the two bottom rows. When HA was injected on Day 4 after the TGFbeta1 but before treadmill running (Figure 2, TTR+HA group), a marked protection against the development of these overgrowths and cartilage erosion at all sites was observed. However, when saline was injected instead of HA (Figure 2, TTR+SA) there was no protective effect on macroscopic pathology.

To further evaluate cartilage protective effects of intra-articular HA injection in the TTR model, Safranin O-stained histological sections were prepared from the knees of six mice from each of the naive, TTR and TTR+HA groups. Typical images of the femoral and tibial surfaces are provided in Figure 3A. The TTR group generally showed thinner and less intensely stained cartilage than the naïve and TTR+HA groups, consistent with the macroscopic findings. When the mean volume of cartilage in the knee joints of mice from the four different groups (Naïve, TTR, TTR+HA and TTR+SA) was estimated by SafraninO staining (see Methods), the TTR model resulted in an approximately 70% loss of cartilage compared to naïve mice (*P *= 0.002) and injection of HA (TTR+HA) largely prevented this loss, whereas with injection of saline (TTR+SA) the loss remained (*P *= 0.001) (Figure 3B).

### Effect of HA injection on acute and long-term histopathological changes in perimeniscal synovium

**Figure 4:**
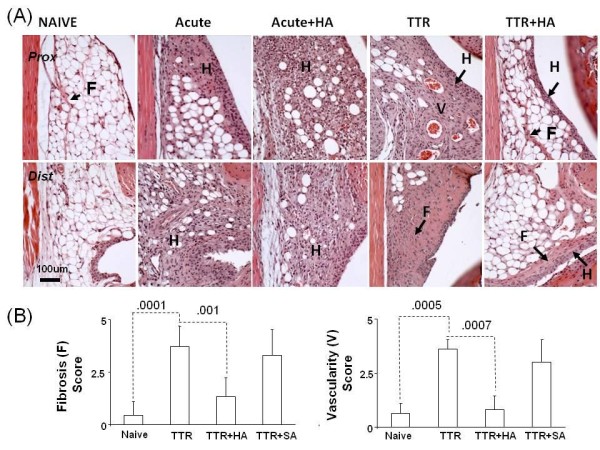
**Effect of injection of HA on synovial histology at Day 4 and Day 19 of model**. (**A**) Typical images of proximal (Prox) and distal (Dist) regions of the perimeniscal synovium from Hematoxylin&Eosin stained sections are shown for five experimental groups (Naïve, Acute, Acute+ HA, TTR and TTR+HA). Histological changes were identified as: hyperplasia (H), fibrosis (F) and neovascularization (V). (**B**) Mean pathology scores for fibrosis (F) and vascularization (V) were obtained from 16 stained sections per mouse (*n *= 4 mice for each of the four experimental groups Naïve, TTR, TTR+HA, TTR+SA) and statistical analysis performed as described in the Methods section.

The effect of HA on the response of synovial tissue to TGFbeta1 (Acute) and TTR was also studied, with four mice in each treatment group, and typical H/E-stained sections from both sets are shown in Figure 4A. A cellular hyperplastic response (H) of both lining and stromal cells was induced by TGFbeta1 injection alone (Acute) and HA injection (Acute +HA) had little or no effect on this early process. After treadmill running, the synovium exhibited extensive fibrotic deposits (F) and these were also frequently populated by multiple blood vessels (V). HA injection (TTR+HA) essentially prevented the fibrotic response in the stromal region and the lining, blocked the vascular response and restored the adipocyte-rich naive appearance of the stroma. When naive mice were run on the treadmill for two weeks there was no sign of changes in the perimeniscal synovium (not shown). To provide quantitative data on these effects between the different treatment groups, we applied a multi-parameter scoring system (see Methods) to each section and the mean +/-SD data (*N *= 4) are provided on Figure 4B. This showed clearly that injection of HA on Day 4 essentially prevented the appearance of fibrotic remodeling and vascular changes by Day 19, whereas saline injection was ineffective in this regard.

### Effect of HA injection on expression of chondrogenic and fibrogenic genes in cartilage/subchondral bone

Our previous studies of this model showed that alterations in macroscopic, chondrogenic and fibrotic tissue responses most readily explained the effects of Adamts5 ablation [16] and HA injection [25]; however, we did not assess specific cellular responses in those studies. Here we have determined expression levels of the chondrogenic genes *Acan*, *Col2a1 and Col10a1 *and the fibrogenic genes *Col1a1, Col3a1 and Col5a1 *in all experimental groups (Naive, Acute, TTR, TTR+HA and TTR+SA) with the tissues separated into cartilage/subchondral bone and meniscus/synovium. This showed that TGFbeta1 injection alone increased the cartilage/subchondral bone expression of *Acan *(2.39-fold) and *Col2a1 *(5.77-fold), whereas *Col10a1 *was essentially unaffected (Table 1). The increased expression of *Acan *and *Col2a1 *was normalized somewhat after the complete TTR, but it was activated, beyond the levels seen with TGFbeta1 alone, in the TTR+HA group. TGFbeta1 injection alone also increased the expression of *Col1a1*, *Col3a1 *and *Col5a1 *by 36.5-fold, 2.97-fold and 2.80-fold, respectively (Table 2 Acute). With TGFbeta1 injection and treadmill running (TTR) the high level of *Col1a1 *expression at Day 5 (Acute group) was maintained, and *Col3a1 *and *Col5a1 *expression was further stimulated to 13.2-fold and 17.9-fold relative to naive levels. Injection of HA (TTR+HA) returned *Col1a1, Col3a1 *and *Col5a1 *expression to near naive levels, whereas injection of saline (TTR+SA) did not markedly affect the enhancement of fibrogenic gene expression seen in TTR. Taken together, the data in Tables 1 and 2 show that a single injection of HA on Day 4 of the TTR model (see Figure 1A) maintains chondrogenic gene expression to Day 19, and also markedly reduces the high level of fibrogenic gene expression seen at this time without HA injection.

**Table 1:** QPCR of chondrogenic gene expression in cartilage/subchondral bone

	acan	*P*	Fold^4^	col2a	*P*	Fold^4^	col102a	*P*	Fold^4^
	ΔCT^1^			ΔCT^1^			ΔCT^1^		
**Naive**	3.84 (0.30)		**1.00**	4.29 (0.25)		**1.00**	6.33 (0.08)		**1.00**
**Acute**	2.58 (0.04)	.0001**^2^**	**2.39**	1.76 (0.38)	.0001**^2^**	**5.77**	6.74 (0.39)	NS**^2^**	**0.75**
**TTR**	3.59 (0.34)	NS**^2^**	**1.90**	2.82 (0.60)	.0014**^2^**	**2.77**	6.59 (0.42)	NS**^2^**	**0.83**
**TTR+HA**	2.13 (0.24)	.0001**^3^**	**3.27**	0.86 (0.19)	.0003**^3^**	**10.7**	4.82 (0.30)	.0001**^3^**	**2.85**
**TTR+SA**	3.36 (0.03)	NS**^3^**	**1.40**	2.32 (0. 4)	NS**^3^**	**3.92**	6.16 (0.43)	NS**^3^**	**1.12**

**^1^**relative to GAPDH, mean (SD) ΔCT of 8 pools with 2 mice per pool for Naïve and 12 pools with 2 mice per pool for all 4 treatment groups.

*P-*values: **^2^**Acute or TTR vs Naive; **^3^**TTR+HA or TTR+SA vs TTR;

**^4^**Fold Changes are relative to Naive

NS, not significant

**Table 2:** QPCR of fibrogenic gene expression in cartilage/subchondral bone

	*col1a*	*P*	Fold^4^	*col3a*	*P*	Fold^4^	*col5a*	*P*	Fold^4^
	ΔCT^1^			ΔCT^1^			ΔCT^1^		
**Naive**	-0.84 (0.23)		**1.00**	6.67 (0.17)		**1.00**	9.98 (0.36)		**1.00**
**Acute**	-6.03 (0.29)	< .0001**^2^**	**36.5**	5.10 (0.71)	.003**^2^**	**2.97**	8.50 (0.41)	.012**^2^**	**2.80**
**TTR**	-6.02 (1.03)	< .0001**^2^**	**36.2**	2.95 (0.44)	.0001**^2^**	**13.2**	5.82 (1.02)	.001**^2^**	**17.9**
**TTR+HA**	-2.44 (0.52)	.0002**^3^**	**3.03**	5.08 (0.59)	.0012**^3^**	**3.03**	7.20 (0.44)	.029**^3^**	**6.87**
**TTR+SA**	-7.25 (1.84)	NS**^3^**	**84.4**	3.15(1.09)	NS**^3^**	**11.5**	4.87 (0.65)	NS**^3^**	**34.5**

**^1^**relative to GAPDH, mean (SD) ΔCT of 8 pools with 2 mice per pool for Naïve and 12 pools with 2 mice per pool for all 4 treatment groups.

*P-*values: **^2^**Acute or TTR vs Naive; **^3^**TTR+HA or TTR+SA vs TTR;

**^4^**Fold Changes are relative to Naive

NS, not significant

### Effect of HA injection on chondrogenic and fibrogenic proteins in cartilage/subchondral bone

**Figure 5:**
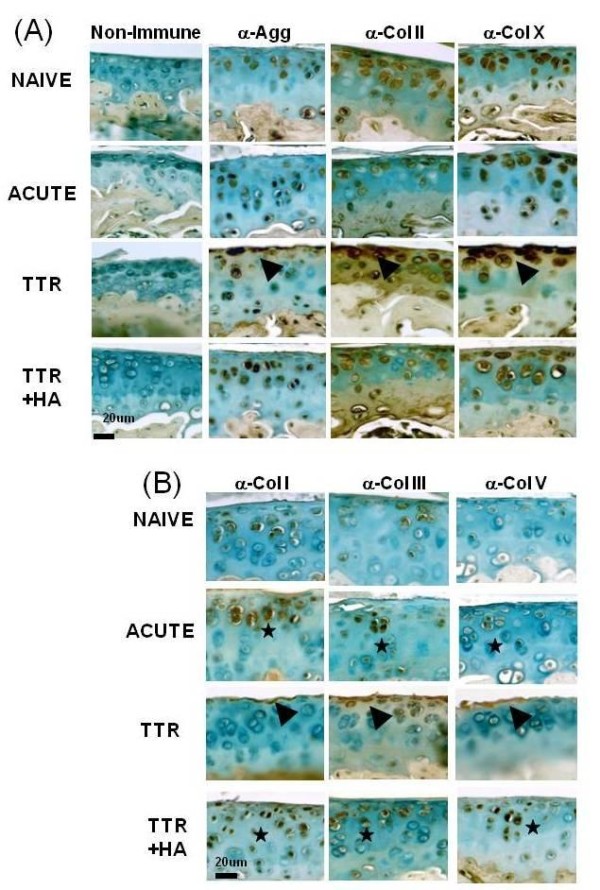
**Immunohistochemical analyses of chondrogenic and fibrogenic matrix molecules in cartilages of naïve and treated mice**. Typical examples of immunostained sections for each antibody, and a non-immune isotype control are shown for the four experimental groups (Naïve, Acute, TTR and TTR+HA). Details of antibodies and staining conditions used are described in the Methods Section. (A) Enhanced staining for chondrogenic proteins (A) and fibrogenic proteins (B) in Acute and TTR+HA conditions are highlighted by black stars, and staining in lesional area of the TTR condition are marked by black arrowheads.

To confirm translation into protein of the chondrogenic genes (*Acan*, *Col2a1, and Col10a1) *and fibrogenic genes (*Col1a1*, *Col3a1, Col5a)*, immunohistochemistry was performed on 12 sections from different sagittal planes of the right lateral tibial compartment of each mouse and typical results are shown in Figure 5. In general agreement with the gene expression data, TGFbeta1 injection alone (Figure 5A, Acute) did not markedly alter the number or location of chondrocytes which were stained for aggrecan, collagen type II and collagen type X. For the TTR group, the degenerated cartilage (note roughened surface) contained cells which were not stained, and also those which were intensely stained in the pericellular/cell-associated space, for aggrecan, collagen type II and collagen type × [28]. Notably, cells staining for these chondrogenic proteins were also frequently seen in the calcified cartilage and subchondral bone regions in all experimental groups that had received TGFbeta1 injections. In the TTR+HA group, the cartilage surfaces remained largely intact and appeared similar to naïve cartilage. However, the underlying chondrocytes showed greatly enhanced pericellular/cell-associated staining for aggrecan when compared to naïve cartilage, a result which is in keeping with the 3.27-fold increase in *Acan *gene expression in cartilage/subchondral bone samples from HA treated joints (Table 1).

As suggested by the gene expression data, TGFbeta1 injection alone also increased the proportion of chondrocytes with pericellular/cell-associated staining for collagen types I, III and V, most of them localized in groups in the mid zone of the articular cartilage (Figure 5B, Acute). Consistent with high gene expression levels for the TTR group, strong staining for collagen types I and III (and to some extent collagen type V) was almost exclusively localized to cells at the surface of the lesions and in the surrounding matrix, whereas chondrocytes in the deeper layers and the calcified cartilage showed only minimal staining for the fibrogenic collagens. Notably, and consistent with the gene expression data, HA injection (Figure 5B, TTR+HA) was accompanied by cell groups which stained for collagen types I, III and V in the protected articular cartilages. Their presence in the mid zone suggests that they represent those cells which were activated by TGFbeta1 in the acute phase.

### Effect of HA injection on expression of chondrogenic and fibrogenic genes in meniscus/synovium

Injection of TGFbeta1 alone resulted in a robust activation (up to 40-fold) of all three chondrogenic genes in the meniscus/synovium (Table 3 Acute), which is in marked contrast to the minimal effect of TGFbeta1 on chondrogenic genes in cartilage/subchondral bone (Table 1 Acute). This difference may be explained by proliferation of stromal cells in the synovial lining (see Figure 4) together with their TGFbeta1-induced differentiation towards a chondrogenic phenotype [29]. For the TTR group, (Table 3 TTR), expression of the three chondrogenic genes remained elevated in the meniscus/synovium pool whereas in the TTR+HA group (Table 3), chondrogenic gene expression was much lower and only minimally above that seen in the naïve joints. Saline injection (Table 3 TTR+SA) did not, however, reduce chondrogenic gene expression in this tissue pool to naïve levels. TGFbeta1 treatment alone (Table 3 Acute) activated *Col1a1 *and *Col5a1 *expression (102-fold and 4.08-fold respectively), but there was no effect on *Col3a1 *expression. For the TTR group, the expression of all three fibrogenic collagen genes was markedly enhanced (15- to 30-fold), much as observed for these genes in cartilage/subchondral bone from the same group. HA, but not saline injection, decreased expression of all fibrogenic genes in the meniscus/synovium tissue pool to essentially naïve levels (Table 3 TTR+HA).

**Table 3:** QPCR of chondrogenic and fibrogenic gene expression in meniscus/synovium

	*acan*	Fold^2^	*col2*a	Fold^2^	*col10a*	Fold^2^	*col1a*	Fold^2^	*col3a*	Fold^2^	*col5a*	Fold^2^
	ΔCT^1^		ΔCT^1^		ΔCT^1^		ΔCT^1^		ΔCT^1^		ΔCT^1^	
**Naive**	3.28	**1.00**	3.73	**1.00**	5.50	**1.00**	-0.21	**1.00**	0.05	**1.00**	3.46	**1.00**
**Acute**	-0.89	**17.5**	-1.59	**39.9**	1.92	**11.9**	-6.89	**102**	-0.13	**1.13**	1.43	**4.08**
**TTR**	0.79	**5.61**	-1.41	**35.3**	1.35	**17.7**	-4.90	**25.8**	-3.66	**13.1**	-1.40	**29.0**
**TTR+HA**	2.99	**1.22**	2.63	**2.10**	4.76	**1.67**	-1.45	**2.43**	-1.11	**2.23**	2.12	**2.53**
**TTR+SA**	1.10	**4.53**	-1.25	**31.5**	1.46	**16.4**	-6.10	**59.3**	-2.68	**6.63**	-0.98	**21.7**

**^1^**relative to GAPDH, mean (SD) ΔCT of 2 pools with 8 mice per pool for Naïve and 2 pools with 12 mice per pool for all 4 treatment groups. Values represent the average of the two pools, with the difference between duplicates being < 20% of average pool value.

**^2^**Fold Changes are relative to Naive

### Effect of HA injection on the abundance of chondrogenic and fibrogenic proteins in the perimeniscal synovium

**Figure 6:**
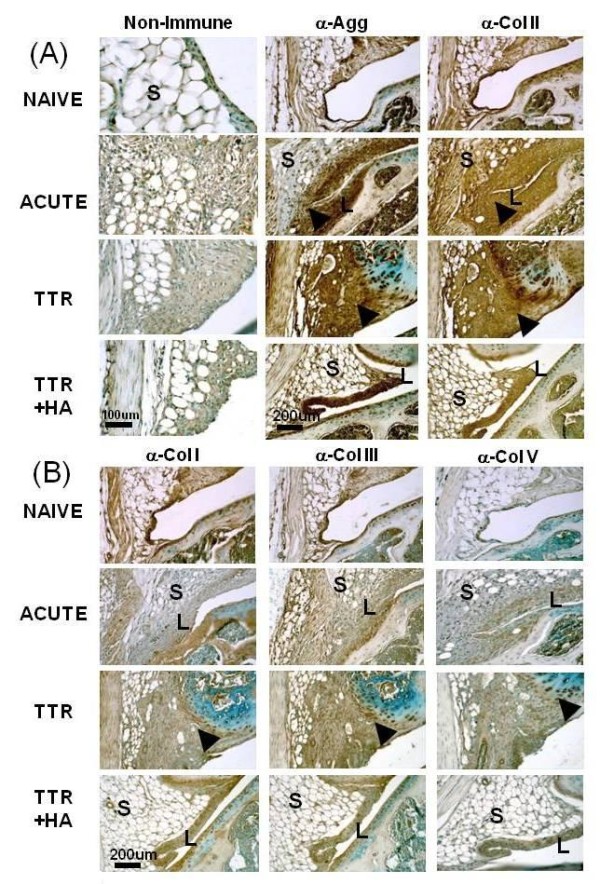
**Immunohistochemical analyses of matrix components in the lateral perimeniscal synovium of naïve and treated mice**. Typical examples of immunostained sections for each antibody, and a non-immune isotype control are shown for the four experimental groups (Naïve, Acute, TTR and TTR+HA). Enhanced staining of aggrecan and collagen II in the hyperplastic (Acute and TTR+HA) and fibrotic regions (TTR) are indicated by black arrow heads. For the TTR condition, strong staining with anti-collagen II or V was seen in the fibrotic regions, as well as around groups of meniscal fibrochondrocytes, and vascular elements stained positively with anti-collagen V. In all treatment groups, collagen X staining patterns were essentially identical to those seen for collagen II (data not shown). L, Lining; S, Stroma.

In naïve joints, the synovial lining and stromal cells showed staining for aggrecan and collagen types I, II and III but not collagen type V (Figure 6). Consistent with the activation of gene expression (Table 3), the TGFbeta1-induced hyperplastic lining (L) showed a strong staining for aggrecan and collagen type II (Figure 6A, Acute). The hyperplastic stromal cell population and its associated matrix also stained for collagen type II, but only weakly for aggrecan. The fibrotic tissues in the synovium that developed in the TTR group were also positive for aggrecan and collagen type II (Figure 6A, black arrow heads), with the latter being particularly strong in the extracellular matrix at the synovial/meniscal interface. In the HA injected joints (TTR+HA) both, cells and matrix in the synovial lining stained strongly for both collagen type II and aggrecan, but in keeping with reduced gene expression (Table 3) only a few cells in the restored adipose tissue-rich stroma showed the presence of these two matrix proteins, closely resembling the staining pattern of the naive stroma (Figure 6A).

Staining for fibrogenic collagens III and V was slightly enhanced in the hyperplastic lining cells; however, the 102-fold acute activation of expression of *Col1a1 *(Table 3) was not seen as enhanced staining for collagen type I in synovium (Figure 6B, Acute) or menisci (data not shown), suggesting that *Col1a1 *transcripts are inefficiently translated or that the newly synthesized protein may not be efficiently cross-linked and incorporated into synovium, but instead diffuse into the synovial fluid.

As expected, the fibrotic tissue deposits seen in the perimeniscal synovium of the TTR group were robustly stained for collagen type III, and to a lesser extent collagen type I, which was mostly concentrated at the meniscus/synovium interface, just like aggrecan and collagen type II (Figure 6A). The vascular elements present in TTR samples (Figures 4A and 6) also stained positive for collagen type III and V but in the TTR+HA group, all three fibrogenic proteins were found only in the synovial cells. Further, the alterations in staining for collagen type III and V in these samples were consistent with the observed decrease in fibrogenic gene expression (Table 3).

### Effect of HA injection on the expression and abundance of ADAMTS5 and MMP13 in cartilage/subchondral bone and meniscus/synovium

The metalloproteinases ADAMTS5 and MMP13 are investigated widely due to their apparent central role in murine OA [16,30], and perhaps in human OA [18,31]. Expression of both A*damts5 *(Table 4) and *Mmp13 *(Table 5) was detectable in both tissue pools for naïve mice, and the expression of both was increased markedly in cartilage/subchondral bone (approximately 6-fold) and meniscus/synovium (approximately 50-fold) in the TTR group, consistent with tissue remodeling. Notably, HA injection (TTR+HA), essentially maintained the low expression levels of both *mmp13 *and *Adamts5 *at the low levels seen in the naïve tissues, whereas saline injection (TTR+SA) was clearly ineffective in this regard.

**Table 4:** QPCR of Adamts5 expression in cartilage/subchondral bone and meniscus/synovium

	CARTILAGE/SC BON			MENISCUS/SYNOVIUM	
	*Adamts5*	*P*	Fold ^5^	*Adamts*5	Fold^5^
	ΔCT^1^			ΔCT^1^	
**Naive**	8.85 (0.55)		**1.00**	8.31	**1.00**
**TTR**	5.94 (0.44)	.0017**^3^**	**7.51**	1.90	**85.1**
**TTR+HA**	7.56 (0.86)	.041**^4^**	**2.44**	5.35	**7.84**
**TTR+SA**	6.22 (1.24)	.009**^4^**	**6.20**	-0.17	**357**

**^1^**relative to GAPDH, mean (SD) ΔCT of 8 pools with 2 mice per pool for Naïve and 12 pools with 2 mice per pool for all 4 treatment groups.

**^2^**relative to GAPDH, mean (SD) ΔCT of 2 pools with 8 mice per pool for Naïve and 2 pools with 12 mice per pool for all 4 treatment groups. Values represent the average of the two pools, with the difference between duplicates being < 20% of average pool value. *P-*values: **^3^**TTR vs Naive; **^4^**TTR+HA or TTR+SA vs TTR;

**^5^**Fold Changes are relative to Naive

**Table 5:** QPCR of mmp13 expression in cartilage/subchondral bone and meniscus/synovium

	CARTILAGE/SC BONE			MENISCUS/SYNOVIUM	
	*mmp13*	*P*	Fold ^5^	*mmp13*	Fold^5^
	ΔCT^1^			ΔCT^1^	
**Naive**	4.74 (0.9)		**1.00**	14.10	**1.00**
**TTR**	1.95 (1.01)	.004**^3^**	**5.73**	9.56	**23.3**
**TTR+HA**	3.50 (1.11)	.045**^4^**	**1.95**	13.15	**1.93**
**TTR+SA**	1.12 (0.75)	.003**^4^**	**12.30**	7.89	**74.4**

**^1^**relative to gapdh, mean (SD) (Delta symbol) CT of 8 pools with 2 mice per pool for Naïve and 12 pools with 2 mice per pool for all 4 treatment groups.

**^2^**relative to gapdh, mean (SD) (Delta symbol) CT of 2 pools with 8 mice per pool for Naïve and 2 pools with 12 mice per pool for all 4 treatment groups. Values represent the average of the two pools, with the difference between duplicates being <20% of average pool value. p values: **^3^**TTR vs Naive; **^4^**TTR+HA or TTR+SA vs TTR; **^5^**Fold Changes are relative to Naive.

**Figure 7:**
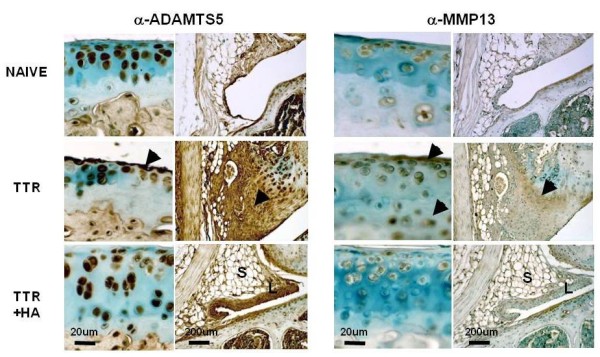
**Immunohistochemical analyses of ADAMTS5 and MMP13 in cartilages and perimeniscal synovium of naïve and treated mice**. (**A**) Immunohistochemical staining patterns form ADAMTS5 and MMP13. Chondrocytes throughout the depth of cartilage showed intense cell-associated staining for ADAMTS5 in all treatment groups. Cells and matrix in hyperplastic (TTR and TTR+HA), or fibrotic (TTR) regions of the synovium and cartilage lesions (TTR) also showed abundant immunoreactive ADAMTS5. (**B**) MMP13 staining was seen only in the pericellular space of chondrocytes in TTR samples, and the matrix at the meniscal/synovial interphase (marked by black arrows).

Immunolocalization of ADAMTS5 in naïve joints showed that it was abundant in a chondrocyte-associated form (but not in matrix) throughout the articular cartilage and also in the synovial lining layer cells and matrix (Figure 7, Naive). This is in keeping with our studies on human cartilages where the enzyme was confined to the pericellular matrix, and in association with HA [27]. For the TTR group, MMP13 stained strongly in chondrocytes and the surrounding matrix at the surface of the cartilage lesions, and also in the underlying subchondral bone cells. In addition, fibrotic tissue in the synovium of these joints stained positive for MMP13 (Figure 7, TTR) and this was particularly noticeable at the interface with the meniscal tissue (black arrow). As predicted from the gene expression studies (Table 5), HA injection prior to treadmill running (TTR+HA) essentially prevented the increase in MMP13 protein abundance seen in the cartilage and synovium of the TTR group. IHC for ADAMTS5 showed that in the TTR group there was a markedly enhanced pericellular staining associated with both flattened cells lining cartilage lesions and underlying cell groups (Figure 7, TTR, black arrow heads). In addition, both cells and matrix were stained throughout the fibrotic regions of the synovium. HA injection before treadmill running (TTR+HA) essentially prevented the increased staining for ADAMTS5 of chondrocytes, and their associated matrix at the cartilage surface. It was notable that for TTR+HA samples, the synovial lining cells and stromal cells in the adipose tissue maintained strong staining for ADAMTS5, whereas mRNA levels in the relevant meniscus/synovium samples were reduced (Table 3).

Lastly, while no statistical evaluation of the meniscus/synovium data (Tables 2, 3 and 5) was possible, the conclusions were based on the following considerations. First, the differences in gene expression between the TTR (or TTR +saline) group and the TTR+HA group was always very marked, being about 4-fold (*Acan*),16-fold (*Col2a1*),10-fold (*Col10a1*),15-fold (*Col1a1*),4-fold (*Col3a1*),10-fold (*Col5a1*),10- to 40-fold (*Adamts5*) and 10- to 30-fold (*Mmp13*). This makes it highly likely that the differences found in tissues pooled from 8 to 12 mice are biologically relevant to the effects of HA. In addition, as described repeatedly in the text above, the changes in expression for each gene evaluated were often supported by changes seen in abundance of the equivalent proteins by IHC.

### Effect of HA injection on the macroscopic pathology seen in CD44 knockout mice

**Figure 8:**
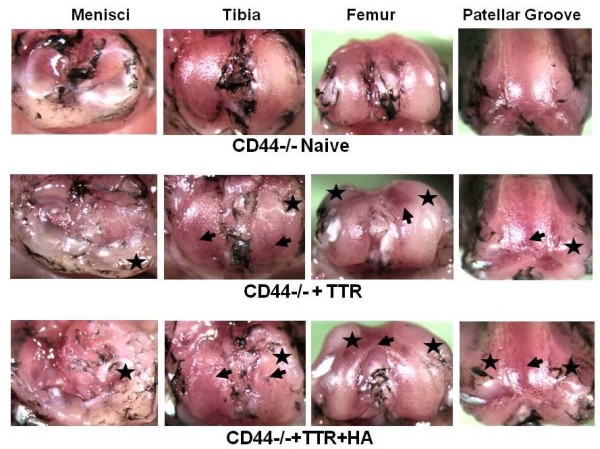
**Menisci and cartilage surfaces of *Cd44*-/- mice show lack of protection by intra-articular HA injections**. *Cd44*-/- male mice were subjected to the TTR model without (*n *= 4) or with (*n *= 4) intra-articular HA injections. Knee joints from those treated mice as well as naïve (*n *= 4) treated mice were examined by India Ink application, photography under a Nikon dissecting microscope (SMZ1000, X6) and images processed with Spot Basic, Diagnostic Instruments, Inc. Typical images of meniscus and cartilage surfaces from naïve joints (top row) and joints from the TTR group (second row) and TTR+HA group (third rows) are shown. Black arrow heads indicate cartilage erosion and black stars indicate fibrotic overgrowth. See Figure 2 for equivalent images from wild-type mice.

While studies with neutralizing CD44 antibodies have implicated CD44 in the inhibitory effects of HA on expression of metalloproteinases by chondrocytes and synoviocytes [19-22], it has also been shown with isolated fibroblasts that their transition to myofibroblasts, a feature of fibrotic remodeling, is modulated by the interaction of CD44 with HA [32]. Since enhanced expression of metalloproteinases and fibrotic remodeling was also seen in joint tissues after TTR, and this was reversed by HA *in vivo*, we decided to further examine the need for CD44 in the protective effects of HA injection. For this purpose we subjected *Cd44*-/- mice [17] to the TTR model and examined the extent to which HA injection was joint protective in the absence of CD44. The macroscopic pathologies seen (Figure 8) clearly indicated that CD44 was essential for the HA effects, suggesting that the binding of the injected HA to cell surface CD44 is essential for its cartilage protection and anti-fibrotic activities in this murine OA model.

## Discussion

The mechanism by which injected HA exerts CD44-dependent anti-fibrotic effects in murine OA appears related to the finding [33] that the fibroblast to myofibroblast transition in progressive murine lung fibrosis is also modulated by HA in a CD44-dependent fashion. In a similar way, it has been shown [34] that HA exhibits a CD44-dependent protection against LPS-induced murine sepsis by binding to TLR4 and blocking excessive inflammatory cytokine production. In this context, we set out to determine whether the cartilage-protective effects of intra-articular HA operate thru a CD44-dependent modulation of the chondrogenic/fibrogenic gene response pathways and/or thru changes in the expression of the critical metalloproteinases, ADAMTS5 and MMP13. Based on the finding that cartilage degradation in the TTR model follows the formation of fibrotic tissue deposits, we hypothesized that it would be associated with the high expression of fibrogenic genes, relative to chondrogenic, and that HA-mediated protection would operate through a reversal to high chondrogenic expression.

To summarize the results (Table 6), we found that in the acute phase of the model (Days 0 to 5), and before evidence of any cartilage lesions, there was a generalized increase in expression of both chondrogenic and fibrogenic genes in both tissue compartments (presumably a direct anabolic response to TGFbeta1 injection). After TTR, and in the presence of tissue fibrosis and cartilage erosion, the chondrogenic genes in both tissue compartments had essentially normalized to naïve levels, except for *Col2a1 *and *Col10a1*, which remained elevated in the meniscus/synovium. At the same time, the fibrogenic genes in both tissue compartments remained elevated or even increased further, particularly in the cases of *Col3a1 *and *Col5a1*. In addition, the expression levels of both Adamts5 and Mmp13 were markedly increased in both tissue compartments in the TTR model. These results are consistent with the idea that cartilage degradation is due to a high expression of fibrogenic genes relative to chondrogenic genes, and also due to a high expression of the metalloproteinases known to be involved in the degradative cascade.

**Table 6:** Summary of tissue changes in the TTR model illustratesthe therapeutic effects of HA injection.

	TTR Relative to Naive	TTR+HA Relative to TTR	TTR+Saline Relative to TTR
**CARTILAGE/SUBCHONDRAL BONE**			

Erosion	+++^1^	--^1^	Unchanged
Chondrogenic Genes	Unchanged	+++	Unchanged
Fibrogenic Genes	+++	---	Unchanged
MMP Genes	+++	---	+

**SYNOVIUM/MENISCUS**			

Perimeniscal Synovitis	+++	--	Unchanged
Chondrogenic Genes	+++	---	Unchanged
Fibrogenic Genes	+++	---	Unchanged
MMP Genes	+++	---	+

**^1^**+/- = 2- to 4-fold Increase/decrease; ++/-- = 2- to 4-fold Increase/decrease; +++/--- = 5- to 10-fold increase

Most importantly, in terms of understanding the mechanism of HA-mediated protection, it was found that HA injection resulted in activation (relative to saline injection) of chondrogenic genes in the cartilage/subchondral bone and a diminution of fibrogenic genes in both tissue compartments. Further, HA injection resulted in a normalization of expression of *Adamts5 *and *Mmp13 *in both compartments. These results indicate that HA-mediated protection is due to a repression of fibrogenesis and an enhancement of chondrogenesis in the cartilage/subchondral bone along with a lowering of the expression of the relevant metalloproteinases in both compartments.

At the histological level, (Figure 6), when TTR samples are examined together with the naïve and acute sections, it is apparent that TGFbeta1 treatment alone results in the appearance of cells with a fibrogenic pericellular/cell-associated matrix, and that during the biomechanical challenge of treadmill running, this cell population is markedly diminished as the surface layer is eroded. However, when HA is injected before treadmill usage (TTR+HA), the fibrogenic pool of cells and associated matrix remains intact. Taken together, the data suggest that HA prevents the catabolic response of chondrocytes which are surrounded by a fibrogenic ECM. In this context, treadmill running alone for 14 days results in only minimal cartilage thinning on the femoral condyles [26] and, although treadmill alone stimulates *Col1a1 *(approximately four-fold) and *Col2a1 *(approximately seven-fold) expression (but not other collagens), this did not cause a detectable accumulation of fibrogenic collagens in the pericellular/cell-associated space (data not shown). It is likely that cartilage erosion in this model is ultimately driven by "catabolic" soluble mediators and one possible source is the mixture of cell types of the remodeled synovial lining. In this context Hematoxylin/Eosin histology of the perimeniscal synovium (Figure 4) showed that HA prevented both fibrotic remodeling and neovascularization, in keeping with the observed inhibition of profibrotic gene expression (Table 3). An associated effect of HA during treadmill running is inhibition of TGFbeta1-induced chondrogenic gene induction in the synovium/meniscus (Table 3), which suggests that the elimination of both fibrotic and chondrogenic cells might promote restoration of the homeostatic adipose-rich stroma of the synovium.

It should be noted that with respect to changes in gene expression, the results were obtained with samples containing more than one type of tissue. In one case it was tibial and femoral cartilage with attached subchondral bone, and in the other it was the lateral and medial menisci with attached synovium. It is, therefore, not possible to discern which cell-types were most affected at the gene expression level by the injection of hyaluronan. However, the immunohistochemical studies did illustrate that in most cases all tissues exhibited protein changes consistent with the alterations in gene expression observed.

A more mechanistic insight into the effects of HA injection seen in this study is suggested by our studies with *Cd44*-/- mice (Figure 8). We have previously reported that CD44, together with pericellular aggrecan and HA can act as a potent inhibitor of profibrogenic TGFbeta1 signaling in dermal wound healing [17]. This could at least in part be explained by a shift from an ALK5/SMAD2,3 (fibrogenic) to an ALK1/SMAD1,5,8 (chondrogenic) signaling response. The requirement for CD44 in HA-mediated protection from joint degradation in this OA model indicates that the injected HA interacts with cell surface CD44 and, thereby, mediates a switch from fibrogenesis to chondrogenesis in the cartilage and adipogenesis in the activated stromal cell population (Figure 4). This hypothesis is strengthened by the knowledge that chondrogenesis and adipogenesis are known to be promoted by strong BMP/ALK1/Smad1/5/8 signaling [35].

### A proposed molecular mechanism for joint protection by HA

The current study shows that HA injection, when used soon after a joint insult, can inhibit the cascade of OA-like changes which occur in the cartilage/subchondral bone and meniscus/synovium (the data are summarized in Table 6). This protective effect of HA has also been demonstrated convincingly in a range of other animal models of OA [3-7,36]. However, uncovering the central process by which HA operates will clearly require further work to delineate its effects on the fate of proliferated synovial stromal cells and also on soluble mediator production during biomechanical stimulation. For example, HA may prevent the CD44-dependent transition of TGFbeta1-activated stromal cells to a stable myofibroblastic phenotype, much as demonstrated in human fibroblast differentiation [37,38]. Indeed, such a process might prevent the appearance of contractile myofibroblasts in human OA cartilage [39]. In addition, HA may reduce apoptosis [40], which in turn could reduce fibrotic remodeling by modulating innate immune responses, as shown for alveolar epithelial cells in lung fibrosis [41].

## Conclusions

We speculate that any beneficial effects of HA injection on pain and function in OA patients [1] result from a normalization of the synovial content of pro-inflammatory and pro-catabolic mediators, which appear to be responsible for cartilage erosion in human OA [42-45]. Such a pathway is consistent with the finding that patient benefit from HA injection appears to depend on the stage and/or sub-type of OA being treated [1,46,47]. Since the therapeutic effects of HA injection described here, and elsewhere, appear to depend largely on its anti-fibrotic activity it is possible that the combined use of HA and an anti-fibrotic agent might improve efficacy. In this regard, it is probably relevant that dosing of rats with GW788388, an agent which has anti-fibrotic effects through inhibition of ALK5, also results in excessive cartilage matrix deposition in the growth zone [48]. Indeed, this is consistent with our suggestion [49] that inhibition of ALK5 in joint progenitor cells will result in a switch from degenerative fibrosis to reparative chondrogenesis in the articular cartilage. Finally, the lack of protection of HA against macroscopic OA pathology in the *Cd44*-/- mouse, provides the opportunity to examine in future experimentation, which cell types are primarily involved in the HA-CD44 mediated therapeutic responses in the knee joint in this murine OA model.

## Abbreviations

ACLT: anterior cruciate ligament transection; ADAMTS5: A Disintegrin and Metalloproteinase with Thrombospondin Motifs 5; EDTH: ethylenediaminetetraacetic acid; FITC: fluorescein isothiocyanate; HA: hyaluronan; MMP13: matrix metalloproteinase 13; MSCs: bone marrow derived mesenchymal stem cells; OA: osteoarthritis; PBS: phosphate-buffered saline; SA: saline; SC: subchondral; TTR: TGFbeta 1 injection with treadmill running model of murine OA.

## Competing interests

JL, DJG, WA, JV and JDS declare no competing interests. AP has received research funding for this study from Seikagaku Corporation. At the time of submission, JT was a full-time employee at Seikagaku Corporation and holds no stocks and shares in the company. Seikagaku Corporation has provided research funding including payment of the article processing fee.

## Authors' contributions

JL performed mouse colony breeding and maintenance, treadmill running, tissue harvests, histological processing, staining and immunohistochemistry. DJG performed all QPCR analyses, data calculation and interpretation. WA performed experiments to determine *in vivo *clearance of FITC-Supartz. JV developed quantitative RNA extraction methods for cartilage/subchondral bone and meniscus/synovial membrane. JDS performed all statistical analyses of data and manuscript preparation. JT provided input on the overall experimental design with regards to experimental groups and mouse numbers and provided technical expertise on Hyaluronan preparations supplied for this study. AP provided the overall experimental design, performed intraarticular injections, and was responsible for data evaluation, interpretation and manuscript preparation. All authors have read and approved the final manuscript.

## Author information

AP holds the Katz-Rubschlager Presidential Chair in Osteoarthritis Research at Rush University Chicago, IL.
